# Epoxides as the Gateway to High-Level Nitrogen Incorporation in Graphene Oxide

**DOI:** 10.1021/acs.jpclett.6c02507

**Published:** 2026-09-24

**Authors:** Yen-Ting Liu, Guan-Ting Chen, Hong-Han Huang, Shunsuke Shimizu, Hong-Yin Chen, Takeharu Yoshii, Hirotomo Nishihara, Virgilio D. Ebajo, Chun-Hu Chen

**Affiliations:** 1 Department of Chemistry, 34874 National Sun Yat-sen University, Kaohsiung 80424, Taiwan; 2 Institute of Multidisciplinary Research for Advanced Materials, 13101 Tohoku University, 2-1-1 Katahira, Aoba-ku, Sendai, Miyagi 980-8577, Japan; 3 Advanced Institute for Materials Research (WPI-AIMR), Tohoku University, 2-1-1 Katahira, Aoba-ku, Sendai, Miyagi 980-8577, Japan; 4 Department of Chemistry, 63148 De La Salle University, 2401 Taft Avenue, Manila 0922, Philippines; 5 Green Hydrogen Research Center, 34874 National Sun Yat-sen University, Kaohsiung 80424, Taiwan

## Abstract

Nitrogen-doped graphene holds immense promise for electrocatalysis, where surpassing the traditional 2–5 at% nitrogenation ceiling requires strategic control of thermodynamic and kinetic factors. Here, we systematically investigate the dominant thermodynamic and kinetic parameters to reach higher nitrogenation. It is demonstrated that, within the investigated 140–200 °C hydrothermal regime, neither kinetic nor mass-transport limitations matter, in sharp contrast to the prevailing literature. Instead, nitrogenation levels are fundamentally governed by the population of basal-plane epoxide groups, meaning that the starting graphene oxide oxidation degree alone limits ultimate doping efficiency. Optimizing precursor oxidation successfully drives the final nitrogen content to 17.3 at% (N/C = 0.24). Subsequent thermal treatment efficiently converts this grafted nitrogen into lattice-doped configurations dominated by pyridinic N (57%). This work establishes a framework showing that maximizing reactive oxygen sites is the definitive pathway to advanced nitrogen-doped frameworks.

## Graphical abstract

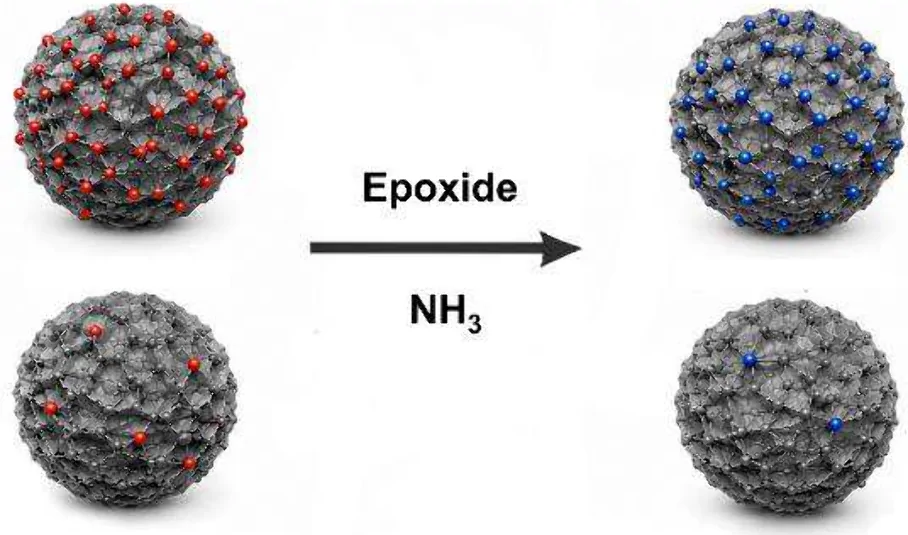

Nitrogen-doped graphene has been widely investigated for electrocatalysis, energy storage, adsorption, and sensing because nitrogen functionalities can modulate the electronic structure and surface chemistry of the carbon framework.^1–5^ Higher nitrogen contents are often associated with improved activity or performance, motivating continued efforts to increase the density of accessible nitrogen configurations at the surface.^6,7^ Despite diverse synthetic strategies, including chemical vapor deposition, thermal annealing, plasma treatment, and hydrothermal or solvothermal processing with ammonia, hydrazine, or urea, the nitrogen contents of nitrogen-doped graphene commonly remain within approximately 2–6 at% and only occasionally reach 7–9 at%.^8–14^ Even solvent-enhanced nitrogenation in our previous graphene composite reached only approximately 7 at%.^15^ The persistence of this nitrogenation ceiling across fundamentally different approaches suggests that the final nitrogen level may not be governed solely by conventional kinetic parameters such as reaction temperature, duration, and nitrogen-source concentration. Mass transport and active-site accessibility have also been proposed as limiting factors because severe restacking of two-dimensional graphene oxide (GO) reduces accessible porosity and restricts the diffusion of nitrogen precursors into interlayer reactive regions. This consideration has motivated the development of expanded or crumpled GO architectures with more open pore structures, although whether enhanced accessibility alone can increase nitrogen incorporation has not been systematically established.^16,17^

A further complication is that the oxidation degree and oxygen speciation of GO precursors are rarely controlled independently of kinetic and structural variables. It therefore remains unclear whether ammonia-based nitrogenation is limited primarily by reaction kinetics, mass transport, or the finite population and intrinsic reactivity of oxygen-containing sites. Because ammonia reacts with oxygen functionalities such as hydroxyl, epoxide, and carboxyl groups, the abundance and chemical identity of these sites may impose an intrinsic upper limit on nitrogen incorporation.^10,18^ Here, we employ a vapor-phase hydrothermal platform to independently examine the effects of reaction temperature, duration, ammonia dosage, precursor porosity, and oxidation degree on GO nitrogenation. GO precursors with controlled oxidation degrees were prepared using a preformed acidic oxidizing medium method, while three-dimensional crumpled graphene oxide generated through a cryogenic process provided a highly open, nonrestacked architecture for evaluating mass-transport and active-site accessibility effects under otherwise matched chemical conditions.^19,20^ The initially introduced surface-associated nitrogen functionalities and their subsequent thermal conversion into lattice-associated pyridinic, pyrrolic, and graphitic configurations were evaluated separately, because the lattice-doped nitrogen contents commonly reported for nitrogen-doped graphene remain relatively modest.^17,21–24^ This design enables kinetic, transport, and oxygen-site effects to be distinguished and provides a framework for identifying the principal factors governing high-level nitrogen incorporation in GO.

To implement this comparison while preventing solvent-induced collapse of CGO, we adopted a vial-in-liner vapor-phase hydrothermal configuration (Figure 1a). Because highly oxidized CGO readily collapses upon direct contact with polar solvents such as water, the GO or CGO powder was placed in a glass vial, while the aqueous ammonia solution was placed outside the vial within a Teflon liner. Nitrogenation therefore proceeded through vapor-phase species diffusing into the vial, eliminating direct liquid contact with the precursor and allowing two-dimensional GO and three-dimensional CGO to be compared without solvent-induced structural collapse.

**Figure 1:**
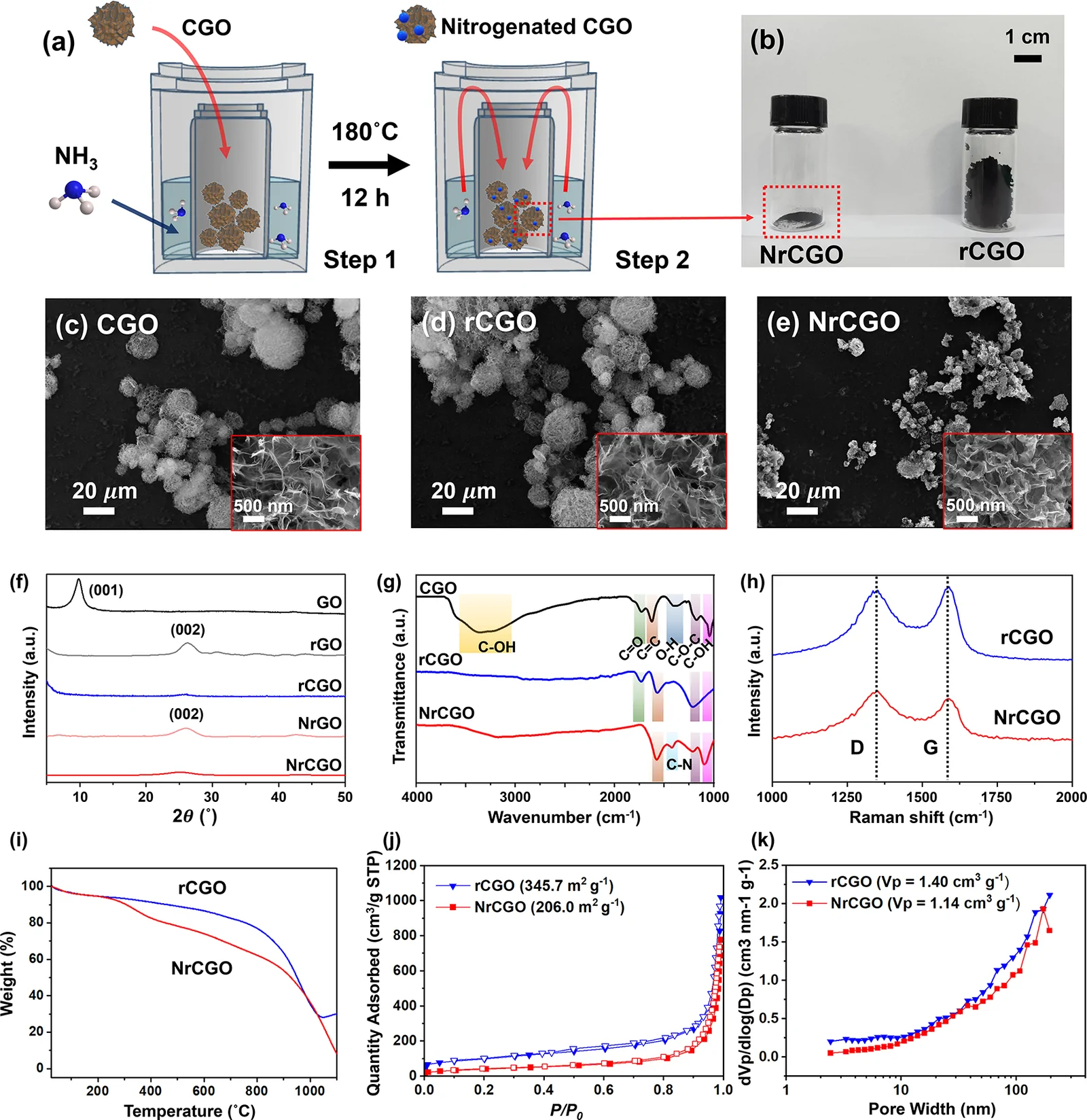
Vapor-phase hydrothermal nitrogenation of crumpled graphene oxide and structural evolution after nitrogen incorporation. (a) Schematic illustration of the vapor-phase hydrothermal nitrogenation process. CGO was placed in a vial inside a Teflon liner to avoid direct contact with the surrounding ammonia solution. During heating at 180 °C for 12 h, NH_3_/H_2_O vapor diffused into the vial and reacted with CGO to form nitrogenated crumpled graphene oxide (NrCGO). (b) Photographs of rCGO and NrCGO with the same sample mass, showing the apparent volume contraction after nitrogenation. (c–e) SEM images of (c) CGO, (d) rCGO, and (e) NrCGO, confirming that the crumpled morphology is retained after vapor-phase treatment and nitrogenation. Insets show the corresponding high-magnification images. (f) XRD patterns of GO, rGO, rCGO, and NrCGO, showing suppressed long-range restacking in the crumpled samples. (g) FTIR spectra of GO, rCGO, and NrCGO, where the emergence of the C–N vibration at approximately 1420 cm^–1^ supports nitrogen incorporation. (h) Raman spectra of rCGO and NrCGO, showing the characteristic D and G bands of graphitic carbon frameworks. (i) TGA profiles of rCGO and NrCGO under N_2_ atmosphere, revealing the different thermal decomposition behavior after nitrogenation. (j) N_2_ physisorption isotherms with the corresponding BET surface areas and (k) BJH pore-size distribution curves of rCGO and NrCGO, indicating that porous features are retained despite partial densification after nitrogenation.

Before assessing these variables experimentally, we established an idealized nitrogenation capacity by treating reactive oxygen functionalities in GO as the available conversion sites. Representative reaction pathways associated with hydroxyl, epoxide, and carboxyl functionalities are schematically illustrated in Scheme 1. Highly oxidized 3 × GO, with an O/C atomic ratio of approximately 0.55 determined by XPS (Table 1), was used as the benchmark for estimating the idealized nitrogenation capacity. Based on literature analyses, the oxygen species in GO are mainly distributed among hydroxyl (∼12%), epoxide/ether (∼34%), and carboxyl (∼27%) species,^25^ but hydroxyl groups are not expected to contribute effectively to nitrogen incorporation. The thermodynamic favorability in terms of free energy and mechanistic plausibility of these representative pathways was evaluated based on the calculated ΔH°, ΔS°, and ΔG° values summarized in Table S1, while the associated functional-group reactivity is further discussed in Supporting Information Section S2. Ketones found on the edge of 3 × GO may also react with NH_3(g)_ to form imines through a carbinolamine intermediate. However, the difficulty of the dehydration step required to convert the unstable carbinolamine intermediate into the corresponding imine under basic conditions renders ketones unlikely to contribute greatly to nitrogen incorporation.^26^ The free energies indicate a clear hierarchy in thermodynamic driving force: nitrogenation at carboxyl- and hydroxyl-derived sites is only modestly favorable (ΔG° in the range of −21.94 to −19.04 kJ mol^–1^), whereas epoxide ring opening is strongly spontaneous (ΔG° ≈ −374.40 kJ mol^–1^). Direct substitution of a C-OH group with either NH_3(g)_ or aqueous NH_4_OH is highly unlikely because hydroxide OH^–^ is a poor leaving group that holds onto carbon too tightly to be displaced, while the excess of water shifts the chemical equilibrium backward, and the formation of unreactive ammonium ions significantly inhibits the reactivity. (see Supporting Information Section S2) Accordingly, epoxide functionalities are expected to constitute the most thermodynamically reactive oxygen sites toward ammonia-based nitrogenation. Based on the measured oxygen content of 3 × GO (O/C = 0.55), we estimated the idealized nitrogenation capacity by assuming complete conversion of all reactive oxygen functionalities according to the stoichiometries proposed in Scheme 1. Hydroxyl groups were excluded from the calculation because they are not expected to contribute significantly to ammonia-based nitrogenation. In contrast, epoxide groups were assumed to undergo a 1O-to-1N conversion through nucleophilic ring-opening by NH_3_,^27^ whereas carboxyl groups were treated as a 2O-to-1N conversion due to ammonium carboxylate formation.^28^ Using the reported oxygen-species distribution in GO and the measured O/C ratio of 3 × GO, the idealized nitrogenation capacity was estimated to be N/C = 0.26, without considering additional carbon loss during hydrothermal treatment. In comparison, applying the same estimation framework to the lower-oxidation commercial GO precursor (O/C = 0.41) yields a lower idealized nitrogenation capacity of approximately N/C = 0.20. Apparently, despite extensive reports on hydrothermal/solvothermal nitrogenation of GO, nitrogen contents approaching this idealized nitrogenation capacity have not been achieved, recognized as the widely observed “nitrogenation ceiling”.^13,29–33^

**Scheme 1:**
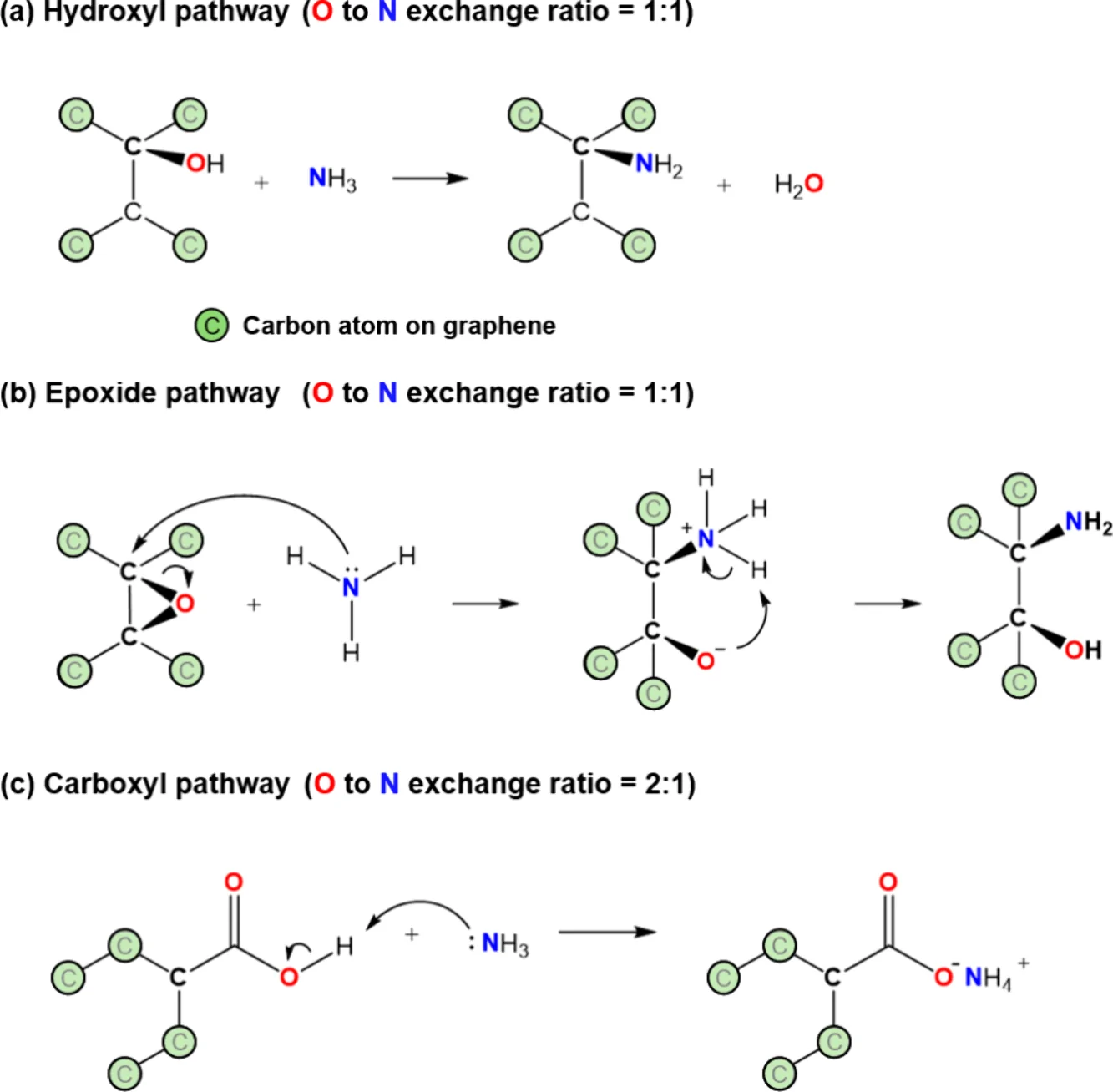
Possible Reaction Pathways and Oxygen-to-Nitrogen Conversion Assumptions for Ammonia-Based Nitrogenation of Oxygen Functionalities in Graphene Oxide^a^

a (a) Possible substitution of basal-plane carbon hydroxyl groups by NH_3_ to form C–NH_2_ motifs with H_2_O elimination; this pathway is unlikely because −OH is a poor leaving group and NH_4_^+^/water suppresses effective amination. (b) Nucleophilic ring opening of epoxide groups by NH_3_, generating amino alcohol functionalities and corresponding to a 1O-to-1N conversion. (c) Acid–base neutralization between edge carboxyl groups and NH_3_, yielding ammonium carboxylate species and corresponding to an effective 2O-to-1N conversion. These stoichiometries were used with the measured oxygen content of 3 × GO to estimate an idealized nitrogenation capacity of N/C = 0.26.

**Table 1:** Effects of Synthetic Parameters on Nitrogen Incorporation

Reaction temp. (°C)^a^	N/C ratio	N (at%)	Reaction time (h)^b^	N/C ratio	N (at%)	NH_3 (aq)_ volume(mL)^c^	N/C ratio	N (at%)	Oxidation degree of GO (O/C)^d^	N/C ratio	N (at%)	GO structure^e^	N/C ratio	N (at%)
140	0.13 ± 0.01	10.9 ± 0.2	6	0.13 ± 0.03	10.7 ± 1.4	2.5	0.15 ± 0.01	12.5 ± 0.6	0.41 (com-mercial GO)	0.16 ± 0.02	12.8 ± 0.9	GO (33.9 m^2^ g^–1^)	0.16 ± 0.01	12.7 ± 0.5
160	0.14 ± 0.01	11.5 ± 0.2	12	0.16 ± 0.01	12.8 ± 0.9	5	0.16 ± 0.03	13.2 ± 2.4	0.49 (1 × GO)	0.15 ± 0.01	12.3 ± 0.2	CGO (330.7 m^2^ g^–1^)	0.16 ± 0.02	12.8 ± 0.9
180	0.16 ± 0.01	12.8 ± 2.7	18	0.15 ± 0.03	11.9 ± 1.8	10	0.16 ± 0.02	12.8 ± 0.9	0.55 (3 × GO)	0.24 ± 0.02	17.3 ± 1.3			
190	0.15 ± 0.02	12.5 ± 0.5	24	0.15 ± 0.01	12.2 ± 0.5	20	0.14 ± 0.02	11.1 ± 1.8						
200	0.16 ± 0.01	13.1 ± 1.0												

a Fixed reaction conditions: reaction time = 12 h, NH_3(aq)_ volume = 20 mL, graphene precursor O/C = 0.41, using CGO as the graphene precursor.

b Fixed reaction conditions: reaction temperature = 180 °C, NH_3(aq)_ volume = 20 mL, graphene precursor O/C = 0.41, using CGO as the graphene precursor.

c Fixed reaction conditions: reaction temperature = 180 °C, reaction time = 12 h, graphene precursor O/C = 0.41, using CGO as the graphene precursor.

d Fixed reaction conditions: reaction temperature = 180 °C, reaction time = 12 h, NH_3(aq)_ volume = 20 mL, using CGO as the graphene precursor.

e Fixed reaction conditions: reaction temperature = 180 °C, reaction time = 12 h, NH_3(aq)_ volume = 20 mL, and graphene precursor O/C = 0.41.

With this idealized nitrogenation capacity in mind, we carried out a one-factor-at-a-time study to quantify the gap between the idealized capacity and experimentally achieved nitrogen incorporation. Specifically, we independently varied kinetic-dependent factors of reaction temperature, reaction time, NH_3(aq)_ dosage, precursor oxidation degree (O/C), and precursor surface area (accessibility/mass transport), while holding all other parameters constant. Unless otherwise specified, all parameter studies were conducted using CGO prepared from commercial GO. Only the oxidation-degree experiments employed different GO precursors with systematically varied oxidation levels. The resulting N­(at%) and N/C ratios are summarized in Table 1. In this work, N­(at%) was calculated from the EA-derived C/N/O contents by converting the weight fractions into molar amounts and normalizing only over C, N, and O, with hydrogen excluded from the calculation. This definition follows the same normalization concept commonly used for XPS-derived atomic percentages, thereby facilitating comparison with reported N­(at%) values in the literature, while the values reported here represent bulk elemental compositions derived from EA rather than surface-sensitive XPS compositions. The detailed calculation method is provided in the Supporting Information. The corresponding elemental compositions (C, O, and N atomic percentages) are summarized in detail in Tables S2 and S3. It should also be noted that elemental analysis of highly hydrophilic GO-based samples may include contributions from residual physisorbed water, which can mainly affect the apparent H and O contents and, in turn, influence the normalized elemental atomic percentages. Because all EA-derived compositions in this work were obtained from samples prepared and analyzed under comparable conditions, this possible contribution does not alter the comparative trends discussed below. TPD–MS provides a useful complementary approach in this regard, because physisorbed species can be removed during thermal desorption, allowing thermally stable and chemically bound nitrogen species to be evaluated more selectively. Further quantitative TPD–MS analysis may therefore provide a more detailed understanding of chemically bound nitrogen contents and nitrogen-species distributions in highly hydrophilic GO-based materials.

We first examined the effect of reaction temperature by scanning the full practical hydrothermal window used in this work (i.e., 140, 160, 180, 190, and 200 °C), with all other reaction parameters kept constant as summarized in Table 1. Because 200 °C approaches the thermal-stability limit of the Teflon liner, no tests were conducted at higher temperatures. The resulting nitrogen contents remain narrowly confined to N/C = 0.13–0.16: 0.13 ± 0.01 (140 °C), 0.14 ± 0.01 (160 °C), 0.16 ± 0.01 (180 °C), 0.15 ± 0.02 (190 °C), and 0.16 ± 0.01 (200 °C) (Table 1). Thus, increasing the hydrothermal temperature from 140 to 200 °C does not further increase the final nitrogen incorporation level. Because epoxide–amine reactions are known to proceed even at substantially lower temperatures,^34^ this reaction pathway is expected to be sufficiently activated within the investigated hydrothermal regime. The nearly constant N/C ratio therefore likely reflects saturation of the finite population of reactive oxygen sites rather than insufficient thermal activation. The nearly unchanged N/C ratio over 140–200 °C is therefore interpreted as a saturation behavior arising from the collective outcome of multiple nitrogenation and deoxygenation steps, rather than as evidence that all elementary nitrogenation processes are temperature-independent. Because the same nitrogenation/deoxygenation pathways are expected to operate throughout this range, the absence of a systematic change in N/C suggests that the final nitrogenation level is largely insensitive to preparation kinetics within the investigated hydrothermal regime. Deoxygenation may also occur concurrently at these temperatures, as thermal removal of oxygen functionalities from GO and amine-functionalized GO has been well-established.^35^ Importantly, oxygen removal alone, such as C–O bond cleavage from amino-alcohol intermediates, does not alter the N/C atomic ratio unless accompanied by carbon loss, because this process does not change the relative numbers of nitrogen and carbon atoms. Based on these results, 180 °C was selected as the standard temperature for subsequent comparisons. This temperature-insensitive behavior in nitrogenation contrasts with several prior reports that attribute higher nitrogen levels to elevated synthesis temperatures.^36,37^

We next evaluated the role of reaction duration at 180 °C by varying the time from 6 to 24 h, yielding N/C = 0.13 ± 0.03 (6 h), 0.16 ± 0.01 (12 h), 0.15 ± 0.03 (18 h), and 0.15 ± 0.01 (24 h) (Table 1). No systematic increase is observed with extended treatment, indicating that the final nitrogen content is likewise time-independent over this range. The resulting N/C values remain within the same narrow plateau range observed in the temperature-dependent study. This conclusion opposes the literature, suggesting that longer reaction times lead to higher nitrogen incorporation in GO.^38,39^ We therefore adopted 12 h as the standard reaction duration.

The effect of nitrogen-source dosage on the nitrogenation level was evaluated by varying the amount of NH_3_ from 2.5 to 20 mL. The resulting nitrogen contents (Table 1) were N/C = 0.15 ± 0.01 (2.5 mL), 0.16 ± 0.03 (5 mL), 0.16 ± 0.02 (10 mL), and 0.14 ± 0.02 (20 mL). Therefore, even an ∼ 8-fold increase in NH_3_ dosage does not increase nitrogen incorporation, and the N/C ratios remain nearly constant and similar to those obtained in the temperature- and time-dependent studies above. We fixed the NH_3(aq)_ volume at 20 mL as an overdose condition in the subsequent experiments to ensure that ammonia availability is not a limiting variable.

By adopting a series of CGO precursors made by GO with varied oxidation degrees, such as O/C = 0.41 of commercial GO, 0.49 of 1 × GO, and 0.55 of 3 × GO, the resulting nitrogen contents are N/C = 0.16 ± 0.02, 0.15 ± 0.01, and 0.24 ± 0.02 (Table 1), respectively. Importantly, only the highly oxidized precursor (O/C = 0.55) produces a clear step-change to N/C = 0.24 ± 0.02, far above the N/C ≈ 0.13–0.16 range observed across all temperature/time/NH_3(aq)_-dosage conditions. In addition, commercial GO (O/C = 0.41) and 1 × GO (O/C = 0.49) yield indistinguishable N/C values within error; it appears that only a significant difference in the GO oxidation degrees can lead to a meaningful shift in the N/C values. These results directly identify precursor oxidation degree as the primary factor governing the final nitrogenation level in this ammonia system.

Finally, we directly tested a widely cited hypothesis that higher surface area and more open porosity enhance accessibility/mass transport and thereby increase nitrogen incorporation.^16,17^ Two precursors with the same O/C = 0.41 but drastically different surface areas, namely GO (33.9 m^2^ g^–1^) and CGO (330.7 m^2^ g^–1^), yield essentially identical nitrogen contents: N/C = 0.16 ± 0.01 (33.9 m^2^ g^–1^) vs 0.16 ± 0.02 (330.7 m^2^ g^–1^) (Table 1). This quantitative control experiment demonstrates that improved accessibility/mass transport alone does not raise nitrogen incorporation when oxidation degree is matched, directly challenging the common assumption that surface area is the key lever for surpassing the nitrogenation ceiling.

Having established the distinct roles of precursor oxidation and accessibility, we next examined the structural consequences of nitrogenation using CGO prepared from commercial GO as a representative model system. CGO was selected because its distinct three-dimensional morphology enables structural retention, densification, and nitrogen distribution to be directly evaluated after vapor-phase treatment. Figure 1b compares nitrogenated crumpled graphene oxide (NrCGO) with the blank control treated under identical conditions in the absence of a nitrogen source, denoted as reduced crumpled graphene oxide (rCGO). NrCGO undergoes appreciable volume contraction, accompanied by an increase in tap density from 1.5 mg mL^–1^ to 100 mg mL^–1^. A small amount of condensate is observed inside the vial, indicating that NH_3_/H_2_O vapor likely condensed during the cooling stage of the preparation process; this condensation is plausibly associated with the observed densification, although the underlying mechanism remains to be clarified. SEM (Figure 1c–e), however, confirms that the wrinkled, crumpled framework is retained after treatment. No obvious large-scale pore formation or fragmentation characteristic of pronounced steam etching is observed at this scale. Overall, rCGO largely preserves the precursor morphology, whereas NrCGO exhibits reduced particle dimensions while retaining a wrinkled and crumpled framework rather than undergoing structural collapse.

Multiple complementary characterizations further support framework preservation and nitrogen incorporation. XRD (Figure 1f) shows that GO exhibits a (001) reflection at 2θ of 9.8°, while rGO and NrGO display broad (002) features near 26°, whereas both rCGO and NrCGO show no obvious layered diffraction, consistent with suppressed long-range intersheet stacking and minimal structural change upon nitrogenation.^40,41^ FTIR (Figure 1g) reveals attenuated oxygen-related bands after treatment and the appearance of a C–N vibration at approximately 1420 cm^–1^ in NrCGO, providing spectroscopic evidence for nitrogen incorporation.^15,42^ Raman spectra of rCGO and NrCGO (Figure 1h) display the characteristic D and G bands of graphitic carbon, indicating that the overall carbon framework is maintained.^43^ TGA (Figure 1i) provides insight into both the thermal stability and the binding nature of the incorporated nitrogen. Relative to rCGO, NrCGO exhibits a noticeably larger mass loss of 5.76 wt% in the 100–300 °C region,^44^ consistent with the decomposition of thermally unstable surface-grafted N functionalities. At higher temperatures, NrCGO continues to show a larger and more sustained weight loss than rCGO, consistent with the gradual decomposition of more stable, lattice-incorporated nitrogen species: the additional loss in 300–1000 °C is commonly associated with lattice-doped N.^45^ Overall, the larger total weight loss and lower residue of NrCGO compared with rCGO indicate that nitrogenation increases the population of reactive functionalities/defects and improves chemical reactivity, but it also reduces thermal stability to some extent.

N_2_ sorption/BJH analyses (Figure 1j,k) indicate that porosity is retained after nitrogenation, although the BET surface area decreases from 345.7 to 206.0 m^2^ g^–1^ with reduced pore volume, consistent with densification. XPS further confirms nitrogen incorporation (Figure 2a,b): an N 1s signal appears at approximately 400 eV, and the N/C atomic ratio reaches about 0.24. Deconvolution of the N 1s spectrum resolves components assignable to pyridinic N, pyrrolic/amine-like N, ammonium N, and oxidized N.^46–48^ HAADF-STEM/EDS mapping (Figure 2c–f) shows homogeneous distributions of C, O, and N throughout the crumpled framework. The combination of retained porosity and abundant, homogeneously distributed nitrogen functionalities also suggests a promising potential in, for example, adsorption and gas capture functionality. In particular, amine-like nitrogen sites in our products should be highly capable of achieving enhanced CO_2_ affinity,^49,50^ whereas protonated/cationic nitrogen sites may facilitate interactions with negatively charged species through electrostatic attraction.^51^ Additional characterization details, spectral analysis, and peak assignments are provided in Section S3 of the Supporting Information.

**Figure 2:**
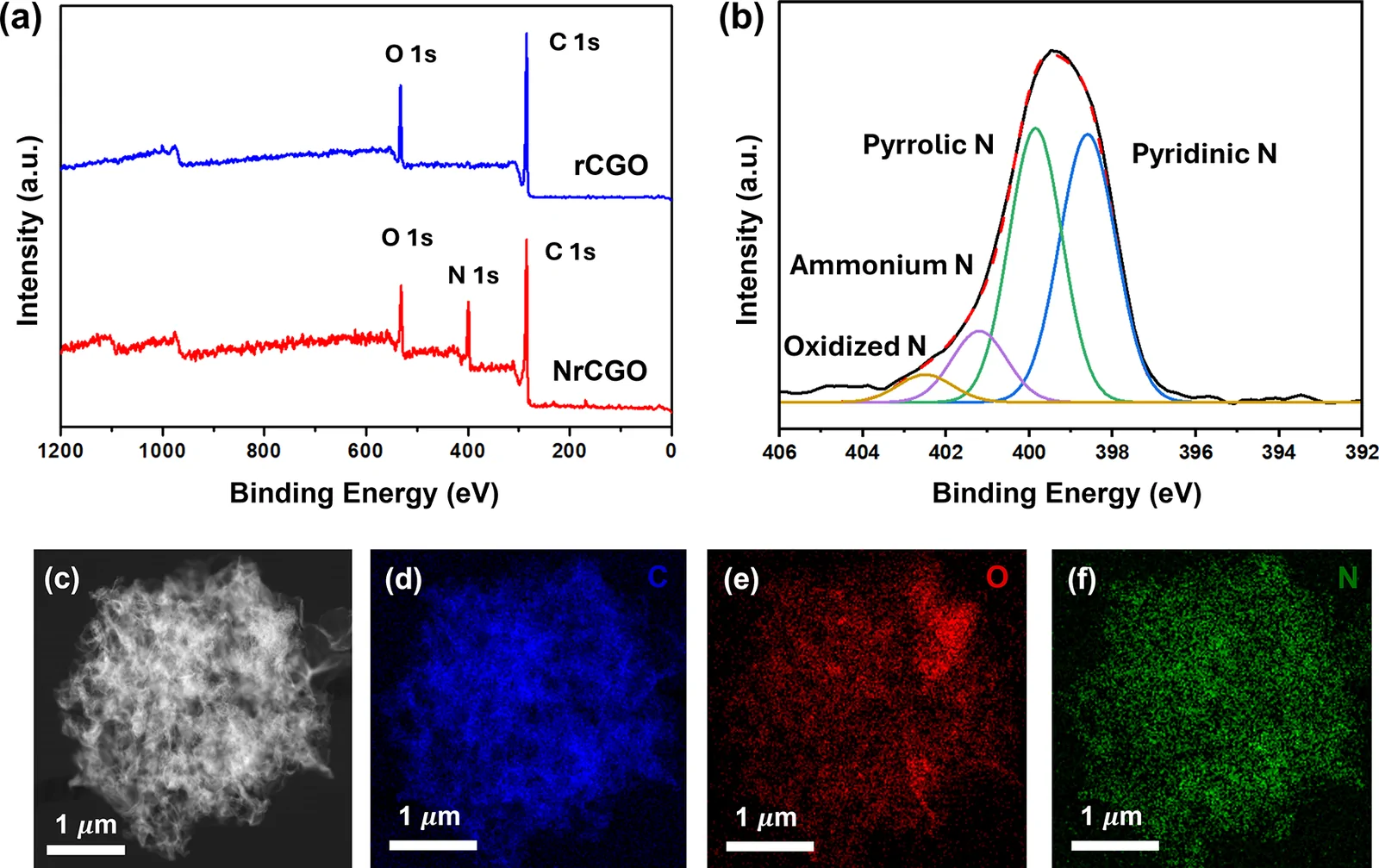
Surface nitrogen speciation and elemental distribution of nitrogenated crumpled graphene oxide. (a) XPS survey spectra of rCGO and NrCGO, showing the emergence of the N 1s signal after vapor-phase nitrogenation. (b) High-resolution N 1s spectrum of NrCGO, deconvoluted into pyridinic, pyrrolic, ammonium, and oxidized nitrogen species. (c) HAADF-STEM image of an individual NrCGO particle, showing the retained wrinkled/crumpled morphology. (d–f) EDS elemental maps collected from the same particle, confirming the homogeneous distribution of (d) C, (e) O, and (f) N throughout the crumpled framework.

With the structural characteristics and parameter-dependent trends established, we next examined the chemical origin of the elevated nitrogen incorporation. When the results in Table 1 are considered collectively, a clear pattern emerges: as long as the precursor oxidation degree remains unchanged, N/C stays near 0.15 despite substantial variations in reaction temperature, duration, ammonia dosage, and precursor accessibility. Compared with the idealized nitrogenation capacity (N/C = 0.20), this plateau suggests that only a subset of oxygen sites can be converted to C–N under our conditions, while certain oxygenated groups remain unreacted even under the most intensive conditions we tested. This finding is distinctive relative to most prior studies, which typically tune one kinetic parameter at a time or adopt higher-porosity architectures without independently matching precursor oxidation degree and oxygen speciation.^21,30^ As a result, the unique N/C plateau can be easily masked. The increase from 1 × GO to 3 × GO is therefore particularly informative because it changes the oxygen-site population and allows us to identify which specific oxygen sites are responsible for pushing nitrogenation beyond the 0.15 plateau.

We first rule out edge carboxyl groups as the dominant contributor: their abundance is inherently constrained by the finite number of edge carbons and therefore cannot increase indefinitely even in highly oxidized 3 × GO.^25,52^ The key evidence comes from nitrogenation with different nitrogen source molecules (Table 2 and Table S4). As shown in Table 2, under identical conditions and using 3 × GO, the nitrogenation degree follows NH_3_ (N/C = 0.24) > CH_3_NH_2_ (N/C = 0.14) > ethylenediamine (N/C = 0.1 after normalization by the two nitrogen atoms in each ethylenediamine molecule), opposite to the basicity trend of ethylenediamine > CH_3_NH_2_ > NH_3_. If the additional sites in 3 × GO were mainly carboxyl groups, stronger bases should either increase nitrogen uptake (via acid–base reaction, which is generally much faster than nucleophilic substitution reactions) or, at a minimum, no decrease trend should be observed. Alternatively, if all these nitrogen sources were sufficiently basic to fully react with carboxyl groups, the final nitrogenation levels would be expected to converge (EDA ≈ CH_3_NH_2_ ≈ NH_3_). This nonconvergence may indicate that nitrogenation on these sites should alternatively be controlled by steric hindrance (ethylenediamine > CH_3_NH_2_ > NH_3_), rather than basicity. Such a proposal is most consistent with the sterically sensitive nature of epoxide groups, where small NH_3_ can access and react more effectively than other bulkier amines, following the order of NH_3_ > CH_3_NH_2_ > ethylenediamine. Therefore, the enhanced nitrogenation observed for highly oxidized GO is attributed to the increased abundance of basal-plane epoxide sites in 3 × GO relative to 1 × GO. This interpretation is further supported by high-resolution C 1s XPS analysis of the three GO precursors (Figure S1 and Table S5). The C–O–C component increases from 28.9% in commercial GO and 30.8% in 1 × GO to 38.9% in 3 × GO, whereas the O=C–O component remains nearly unchanged (3.6–4.4%) and neither C–OH nor C=O shows a corresponding monotonic increase.

**Table 2:** Effect of Nitrogen Source and Post-Annealing Treatment Temperature on the Nitrogenated Crumpled Graphene Oxide.

N-source	N/C ratio	N (at%)	Annealing temp. (°C)	N/C ratio	N (at%)
NH_3_	0.24 ± 0.02	17.3 ± 1.3	300	0.20	14.0
CH_3_NH_2_	0.14 ± 0.01	11.4 ± 0.8	600	0.11	8.54
EDA	0.20 ± 0.01	15.6 ± 0.4	900	0.07	5.72

This interpretation is further supported by the comparison between the theoretical and experimentally achieved nitrogenation levels. While lower-oxidation GO (O/C = 0.41) exhibits a calculated idealized nitrogenation capacity of only N/C ≈ 0.20 and experimentally remains near the commonly observed N/C ≈ 0.15 plateau, highly oxidized 3 × GO shows both a higher idealized capacity (N/C ≈ 0.26) and a substantially higher experimentally achieved nitrogenation level (N/C ≈ 0.24). Previous studies on highly oxidized GO systems showed that additional oxidation preferentially increases basal-plane epoxide populations after edge carboxyl groups approach saturation.^25^ Such behavior is therefore consistent with the proposal that epoxide groups are the dominant reactive sites responsible for pushing nitrogenation beyond the conventional N/C ≈ 0.15 plateau. Comparison with reported nitrogenated graphene systems (Table S6) further shows that conventional ammonia-based hydrothermal nitrogenation of GO typically results in relatively modest nitrogen incorporation levels. Although alternative nitrogen sources and harsher nitrogenation methodologies can also achieve high nitrogen incorporation, the present results highlight precursor oxidation degree as a critical yet previously underemphasized parameter for increasing nitrogen incorporation. Such oxygen-site-guided considerations may also provide useful insight for further optimization of other graphene nitrogenation methodologies. The resulting oxygen-site-dependent nitrogenation mechanism is summarized in Scheme 2.

**Scheme 2:**
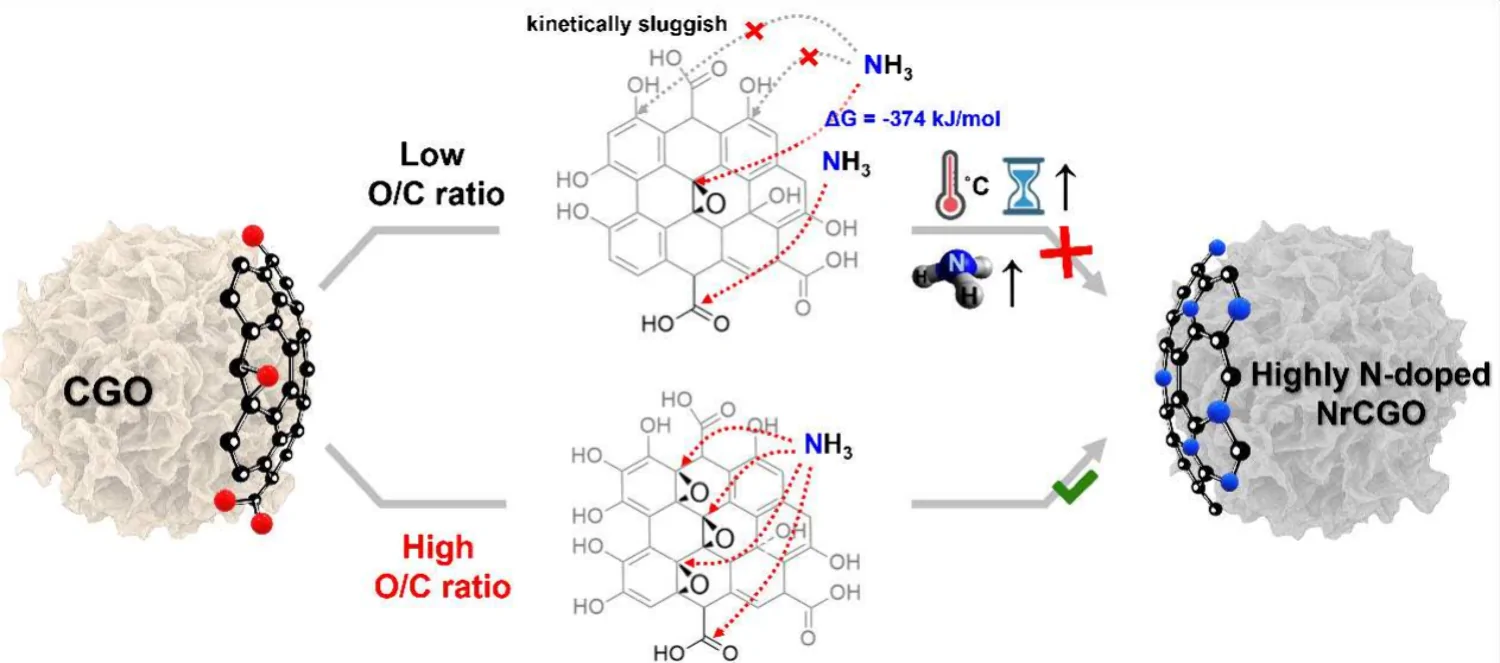
Schematic Summary of Oxygen-Site-Dependent Nitrogenation of Crumpled Graphene Oxide (CGO)^a^

a Nitrogen incorporation is weakly affected by reaction temperature, time, and ammonia dosage but is strongly governed by the precursor oxidation degree. Increasing the O/C ratio, particularly the abundance of epoxide functionalities, provides additional reactive sites and enables the formation of highly N-doped NrCGO.

Having identified epoxide-rich oxygen chemistry as the origin of enhanced nitrogen incorporation, we next examined how the initially introduced nitrogen functionalities evolved during thermal treatment. Elemental analysis of NrCGO annealed at 300, 600, and 900 °C shows a progressive decrease in nitrogen content with increasing temperature (Table 2 and Table S4), indicating that the retained nitrogen level can be tuned through the annealing conditions. The corresponding FTIR spectra of NrCGO after thermal treatment at 300, 600, and 900 °C are provided in Figure S2. This trend indicates the progressive loss of thermally unstable nitrogen species with increasing annealing temperature, while the nature of the retained nitrogen configurations was further examined by TPD–MS and XPS.

We therefore employed TPD–MS as a bulk-sensitive speciation probe and interpreted the NH_3_/HCN/N_2_/NO evolution (Figure 3a-d) using the mechanistic assignment reported by Yoshii et al.^46^ Peak deconvolution (Figure 3e) shows that NrCGO-600 °C is dominated by pyridinic N (57%), followed by pyrrolic N (32%) and a smaller fraction of graphitic N (10%), with oxidized nitrogen negligible (approximately 1%). These results were also confirmed by XPS quantification (Figure 3e). This pyridinic/pyrrolic bias indicates that annealing at elevated temperatures preferentially stabilizes defect-associated sp^2^ nitrogen (commonly assigned to vacancy/hole boundaries or nonhexagonal reconstructions), whereas graphitic N substitution within a more intact basal-plane hexagonal network remains a minority under these conditions. Notably, the pyridinic-N fraction observed here is comparatively high relative to many reported nitrogen-doped graphene/carbon systems (Table S7), which may be advantageous for future catalytic applications.^24,53,54^

**Figure 3:**
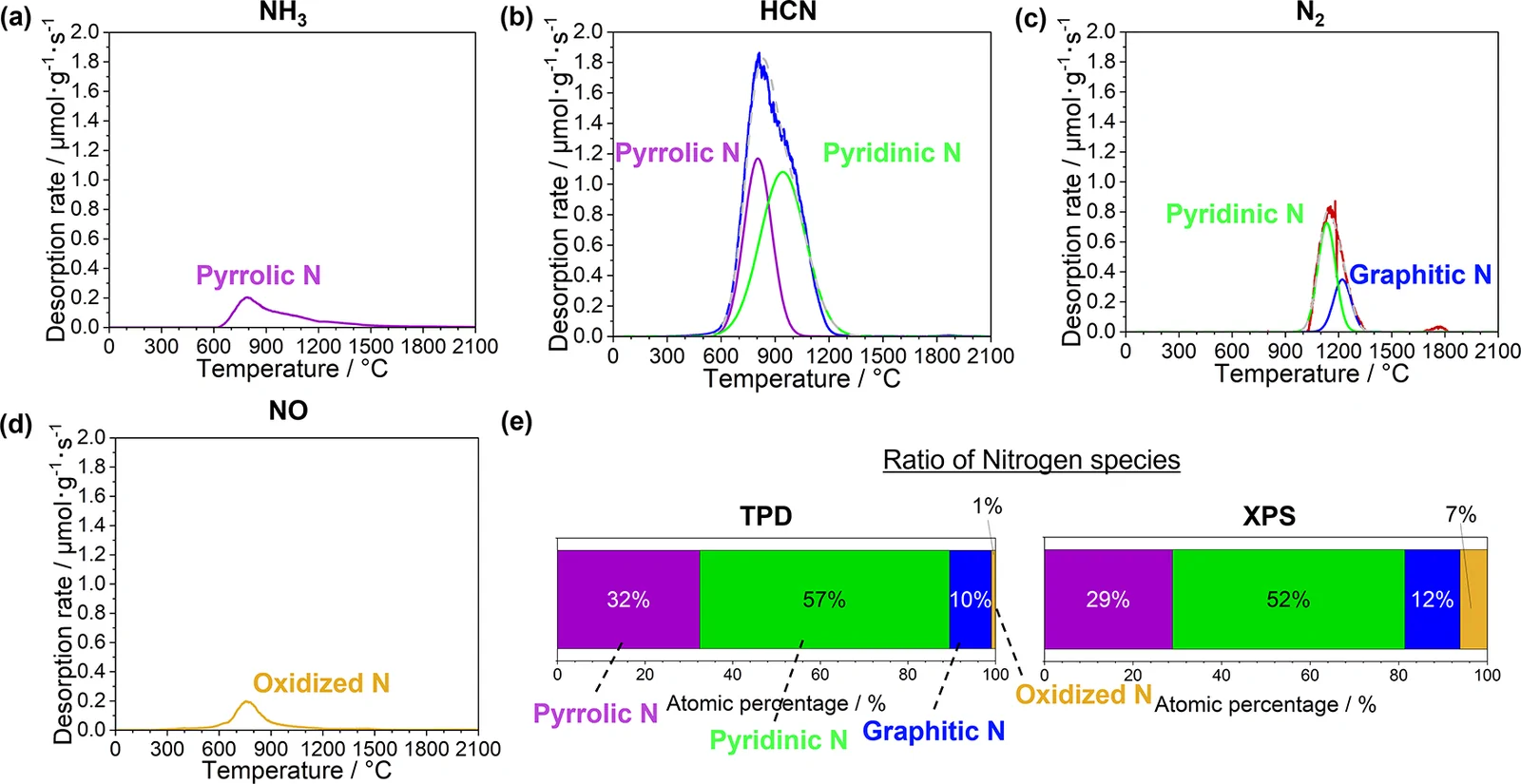
Bulk-sensitive quantification of nitrogen configurations in thermally treated NrCGO. (a–d) TPD profiles of desorbed nitrogen-containing species from NrCGO-600 °C, including (a) NH_3_, (b) HCN, (c) N_2_, and (d) NO. The deconvoluted signals are assigned to pyrrolic, pyridinic, graphitic, and oxidized nitrogen species based on their characteristic desorption behavior. (e) Quantitative distribution of nitrogen configurations derived from TPD analysis, compared with the surface-sensitive XPS results.

Although pyridinic and pyrrolic nitrogen are frequently discussed as “edge-type” motifs,^46,55^ this assignment does not necessarily imply only the outer perimeter of a graphene sheet. In our system, the major nitrogenation pathway originates from basal-plane epoxide mediated by NH_3_-driven ring opening to introduce a local O–C–C–N motif. During annealing and deoxygenation, effective removal of oxygen in every O–C–C–N motif may result in a large quantity of local defects and thus lead to a reconstruction into nonhexagonal rings and vacancy-like defects, which effectively yield edge-like boundaries within the sheet.^56^ In addition, hydrothermal steam etching may also provide a secondary pathway for generating defect-associated sites by preferentially removing defective sp^3^-rich domains and creating nanoscale pores and internal edges. Such steam-induced defects may therefore contribute to the subsequent formation of edge-like nitrogen configurations.^57^ Consequently, the pyridinic-rich signature observed here can be rationalized as arising from both basal plane functional groups and defect-associated reconstruction, not only from macroscopic outer edges.

As a preliminary assessment of functional activity, NrCGO-600 °C was evaluated for the oxygen reduction reaction. Relative to rCGO, NrCGO-600 °C exhibits a 0.08 V positive shift in ORR onset potential (Figure S3), indicating that the nitrogen configurations generated through nitrogenation and subsequent annealing are electrocatalytically active. This result provides functional validation of the incorporated nitrogen species. It is worth noting that ORR performance is governed not only by the total nitrogen content but also by the resulting nitrogen configuration and electrical conductivity.^47,55,58^ Increasing nitrogen incorporation can raise the population of catalytically relevant sites, particularly pyridinic-N-associated defect sites,^47,53^ while excessive defect formation may disrupt the conjugated carbon network and impede electron transport.^59,60^ In addition, different nitrogen configurations influence the electronic structure of graphene differently. Graphitic N is generally more compatible with preservation of the conjugated carbon framework and efficient charge transport,^61^ whereas pyridinic and pyrrolic N are commonly found at edge- and defect-associated sites that can be catalytically relevant.^46^ Therefore, achieving high ORR activity requires a balance among nitrogen content, nitrogen configuration, and electrical conductivity. Further optimization of this balance and selective control of nitrogen configurations using the present nitrogenation strategy remain subjects for future study.

In summary, we have systematically decoupled the chemical and physical factors governing the ammonia-based hydrothermal nitrogenation of graphene oxide, revealing that the widely observed ″nitrogenation ceiling″ in this commonly used system is neither a kinetic nor a mass-transport limitation, but is fundamentally an oxygen-site-limited phenomenon. Through rigorous control experiments using a vapor-phase hydrothermal platform and 3D crumpled architectures, we demonstrated that conventional parameters—including reaction temperature (up to 200 °C), duration, ammonia dosage, and even active-site accessibility—exert negligible influence on final nitrogen levels when the precursor oxidation degree is held constant. Our findings establish that the population of basal-plane epoxide groups serves as the primary gateway for nitrogen incorporation. By modulating the oxidation degree of the graphene precursor, we achieved a nitrogen content of 17.3 at% (N/C = 0.24), among the highest values reported for ammonia-based hydrothermal nitrogenation of GO. Comprehensive characterization via TPD–MS and XPS confirms that these surface-grafted nitrogen species can be effectively converted into stable lattice-doped motifs—predominantly pyridinic nitrogen (57%). Possible additional carbon loss and framework reconstruction during hydrothermal treatment may also affect the final N/C ratio, and their quantitative contributions remain to be clarified in future studies. It should be noted that this conclusion does not exclude the possibility that higher-temperature treatments, different nitrogen sources, or other nitrogenation methodologies rather than hydrothermal-based treatment may lead to different kinetics of nitrogen incorporation. Rather, this work provides a fundamental guideline in the design of highly heteroatom-doped graphene materials via hydrothermal systems, shifting the focus from kinetic optimization to the precise engineering of precursor oxygen speciation. The oxygen-guided nitrogenation strategy presented here not only offers practical knowledge to pursue even higher nitrogen doping levels, but also may open further opportunities for developing high-performance graphene-based materials for energy storage and catalysis.

## Supplementary Material



## Data Availability

The data supporting the findings of this study are available in the main manuscript and the Supporting Information.

## Notes

Experimental procedures for the synthesis of GO, CGO, rCGO, and NrCGO, including vapor-phase hydrothermal nitrogenation and ORR electrode preparation (Section S1); thermodynamic and mechanistic considerations for representative nitrogenation pathways on graphene oxide, including model reactions and calculated thermodynamic parameters (Section S2 and Table S1); supplementary structural and surface characterizations of rCGO and NrCGO by XRD, FTIR, Raman, TGA, BET/BJH, and XPS analyses (Section S3); elemental compositions of nitrogenated crumpled graphene synthesized under different hydrothermal reaction conditions determined by elemental analysis (Table S2); elemental compositions of NrCGO synthesized using graphene oxide precursors with different oxidation degrees and structures determined by elemental analysis (Table S3); elemental compositions of NrCGO synthesized using different nitrogen sources and after annealing at different temperatures determined by elemental analysis (Table S4); abundance percentages of different functional groups on the surfaces of commercial GO, 1 × GO, and 3 × GO based on high-resolution C 1s XPS peak deconvolution (Table S5); comparison of nitrogen incorporation levels among reported nitrogenated graphene-based materials (Table S6); quantification of nitrogen configurations by TPD–MS and comparison with reported nitrogen-doped graphene/carbon materials (Table S7); high-resolution C 1s XPS spectra and peak deconvolution of 3 × GO, 1 × GO, and commercial GO (Figure S1); comparative FTIR spectra of NrCGO and thermally reduced NrCGO samples treated at 300, 600, and 900 °C (Figure S2); LSV curves of rCGO and NrCGO-600 °C in O_2_-saturated 0.1 M KOH (Figure S3) (DOCX)
