# Supplementary material for: Epoxides as the Gateway to High-Level Nitrogen Incorporation in Graphene Oxide
Source: J Phys Chem Lett. 2026 Sep 24;17(44):12680–90. doi: 10.1021/acs.jpclett.6c02507 (PMC13746813; doi:10.1021/acs.jpclett.6c02507)
Supplement: Supplementary file 1 [file jz6c02507_si_001.docx]

Supporting Information

for

**Epoxides as the Gateway to High-Level Nitrogen Incorporation in Graphene Oxide**

Yen-Ting Liu^1^, Guan-Ting Chen^1^, Hong-Han Huang^1^, Shunsuke Shimizu^2^, Hong-Yin Chen^1^, Takeharu Yoshii^2^, Hirotomo Nishihara^2, 3^, Virgilio D. Ebajo, Jr.^4^, and Chun-Hu Chen* ^1, 5^

^1^Department of Chemistry, National Sun Yat-sen University, Kaohsiung, Taiwan 80424

^2^Institute of Multidisciplinary Research for Advanced Materials, Tohoku University, 2-1-1 Katahira, Aoba-ku, Sendai, Miyagi 980-8577, Japan

^3^Advanced Institute for Materials Research (WPI-AIMR), Tohoku University, 2-1-1 Katahira, Aoba-ku, Sendai, Miyagi 980-8577, Japan

^4^Department of Chemistry, De La Salle University, 2401 Taft Avenue, Manila 0922, Philippines

^5^Green Hydrogen Research Center, National Sun Yat-sen University, Kaohsiung, Taiwan 80424

*E-mail: chunhu.chen@mail.nsysu.edu.tw

**S1. Experimental Section**

**S1.1. Synthesis of graphene oxide (GO)**

Graphene oxide (GO) was synthesized using the preformed acidic oxidizing medium approach (PAOM) previously developed in our laboratory.^1^ In a typical preparation, 3 g of potassium permanganate was added to 23 mL of concentrated sulfuric acid and mixed in an ice bath for 3 min. After thorough mixing, 1 g of graphite powder was introduced and stirred for 10 min. The mixture was then transferred to an oil bath at 35°C and stirred for 1 h. Subsequently, 50 mL of deionized water was added in five portions, and the mixture was heated to 85 °C for 15 min. After cooling, 10 mL of hydrogen peroxide was added to obtain a yellow GO dispersion. The product was washed sequentially with hydrochloric acid and acetone, and the resulting material is denoted 1$\times$GO. To obtain GO with a higher oxidation degree, the same PAOM procedure was carried out using three times the amount of KMnO₄ (9 g) while keeping all other conditions identical. The resulting highly oxidized GO is referred to as 3$\times$GO.

**S1.2. Low-temperature roller method for the synthesis of crumpled graphene oxide sphere (CGO)**

Crumpled graphene oxide (CGO) spheres were prepared following our previously reported low-temperature roller protocol.^2^ A 5 mg mL⁻¹ dispersion of GO was first prepared, and the motor-driven roller was activated while liquid nitrogen was poured into a stainless-steel pan to precool both the pan and the roller for 15 min. The GO dispersion was then delivered into an automatic sprayer mounted above the roller. Upon operation, the sprayer atomized the dispersion, and the aerosolized droplets were immediately carried into the liquid nitrogen to form frozen GO microdroplets. The frozen material was subsequently freeze-dried (20 mtorr, -40°C, 11 h) to yield crumpled graphene oxide (CGO). The same procedure was applied to GO samples with different oxidation levels. Using 1$\times$GO as the precursor produced 1$\times$CGO, whereas using 3$\times$GO produced 3$\times$CGO. All roller-based steps were kept identical, ensuring that differences among CGO, 1$\times$CGO, and 3$\times$CGO arise solely from the oxidation degree of their respective GO precursors.

**S1.3. Thermal reduction of GO and CGO**

The as-synthesized GO/CGO was placed in a tubular furnace under an argon atmosphere for 15 min. The temperature was then gradually increased from room temperature to 300°C over 3 h, followed by heating to 600°C within 1 h. The sample was maintained at 600°C for 2 h, and subsequently allowed to cool naturally to room temperature in the furnace, yielding reduced crumpled graphene oxide (rCGO).

**S1.4. Synthesis of NrCGO using ammonium hydroxide**

50 mg of CGO was placed in a sample vial, which was then inserted into a Teflon-lined stainless-steel autoclave. Subsequently, 20 mL of ammonium hydroxide solution was added to the liner. The autoclave was sealed and heated in an oven from room temperature to 180°C over 1 h, followed by maintaining at 180°C for 11 h. After the reaction, the product was washed thoroughly with deionized water and ethanol, and the excess solvent was removed under vacuum, yielding nitrogen-doped reduced crumpled graphene oxide (NrCGO). The synthesis of NrCGO using methylamine and ethylenediamine (EDA) as the nitrogen source followed the same procedure, except that 20 mL of ammonium hydroxide was replaced with the corresponding nitrogen-source solution.

**S1.5. Preparation of rCGO catalyst ink and working electrode**

rCGO/NrCGO was used as the electrocatalyst for oxygen reduction reaction measurements. The catalyst ink was prepared by dispersing 1.0 mg of rCGO powder in 0.88 mL of a mixed solvent composed of deionized water and isopropanol in a volume ratio of 1:1. Subsequently, 2.66 µL of a 5 wt% Nafion solution was added as the binder. The resulting suspension was ultrasonicated for 15 min to obtain a homogeneous ink. A glassy carbon rotating disk electrode (RDE, GC50) was employed as the working electrode. A volume of 34.84 µL of the as-prepared catalyst ink was drop-cast onto the polished glassy carbon surface and dried under ambient conditions, corresponding to a catalyst loading of approximately 200 µg cm⁻². After drying, an additional 15 µL of a diluted Nafion solution (0.05 wt% Nafion in H₂O: isopropanol (IPA) = 1:1 by volume) was drop-cast on top of the catalyst layer to improve film integrity and adhesion. The electrode was then allowed to dry naturally before electrochemical measurements.

**S1.6.** **Measurement of oxygen reduction activity**

Electrochemical measurements were carried out using a conventional three-electrode system equipped with a rotating disk electrode. An Ag/AgCl electrode and a platinum plate were used as the reference electrode and counter electrode, respectively. The electrolyte was a 0.1 M KOH aqueous solution saturated with oxygen. Before each measurement, the electrolyte was purged with O₂ for at least 20 min to ensure full saturation. All electrochemical potentials were converted to the reversible hydrogen electrode (RHE) scale. Cyclic voltammetry (CV) was first performed without electrode rotation within the same potential window used for linear sweep voltammetry to electrochemically clean and stabilize the catalyst surface. Linear sweep voltammetry (LSV) for ORR evaluation was subsequently conducted at a rotation rate of 1600 rpm with a scan rate of 10 mV s⁻¹. The potential range for ORR measurements was 0 to 1.0 V versus RHE. During the measurements, a continuous O₂ flow was maintained above the electrolyte to preserve oxygen saturation.

**S1.7. TPD–MS analysis of nitrogen species**

To quantify the total amount of nitrogen species in the carbon materials, a high-temperature vacuum temperature-programmed desorption (TPD) measurement was performed up to 2100°C with a heating rate of 10°C min⁻¹ using a homemade advanced TPD system. The sample was placed in a graphite vessel inside a quartz chamber and evacuated to a high vacuum below 2×10⁻⁵ Pa. The temperature was increased from room temperature to 2100°C by a high-frequency induction heater, and the desorbed gases were analyzed using a quadrupole mass spectrometer.

The total nitrogen content (*N_total_*) was estimated by integrating the desorption signals of nitrogen-containing species, including N₂, NH₃, HCN, and NO detected during the TPD measurement:

𝑁_total_= *N*_N₂_+ *N*_NH₃_+ *N*_HCN_ + *N*_NO_

where *N*_N₂_, *N*_NH₃_, *N*_HCN_, and *N*_NO_ represent the amounts of desorbed nitrogen-containing gases.

Furthermore, the chemical speciation of nitrogen species was analyzed by correlating the TPD desorption profiles with X-ray photoelectron spectroscopy (XPS) results, following Yoshii *et al.* methodologies.^3^ This combined approach enables the assignment of different nitrogen functionalities, such as pyrrolic-N, pyridinic-N, and graphitic-N, based on the characteristic desorption behavior of each gas species.

**S1.8. Calculation of EA-derived atomic percentages**

The atomic percentages of C, N, and O were calculated from the elemental-analysis-derived C/N/O weight contents. The measured weight fractions of C, N, and O were first converted into molar amounts, and the nitrogen atomic percentage was calculated using the following equation:

$$N \left( at\% \right)=\left[ \frac{\left( \frac{w_{N}}{M_{N}} \right)}{\left( \frac{w_{C}}{M_{C}} \right)+\left( \frac{w_{N}}{M_{N}} \right)+\left( \frac{w_{O}}{M_{O}} \right)} \right]\times100\%$$

where $w_{C}$, $w_{N}$, and $w_{O}$are the weight percentages of carbon, nitrogen, and oxygen obtained from EA, respectively. $M_{C}$, $M_{N}$, and $M_{O}$are the atomic weights of carbon, nitrogen, and oxygen, respectively.

Hydrogen was excluded from this normalization. This definition follows the same elemental-normalization concept commonly used for XPS-derived atomic percentages,^4^ in which hydrogen is not included in the elemental composition. The reported N/C ratios were calculated from the EA-derived atomic percentages.

**S1.9. Material characterization**

Scanning electron microscopy (SEM) images were acquired using an FEI Inspect F50 field-emission scanning electron microscope operated at 5 kV. X-ray diffraction (XRD) patterns were recorded on a Bruker D2 Phaser diffractometer equipped with a Cu Kα radiation source (λ = 1.5418 Å). Nitrogen adsorption–desorption isotherms were measured at 77 K using a volumetric gas sorption analyzer. The Brunauer–Emmett–Teller (BET) surface area was determined from the adsorption branch in the relative pressure range of 0.01–0.05, and the pore size distribution was derived from the desorption branch at a relative pressure of 0.96 using the Barrett–Joyner–Halenda (BJH) method. Raman spectra were collected with a WITec Alpha300R confocal Raman microscope using a 532 nm excitation laser. Fourier transform infrared (FTIR) spectra were obtained with a PerkinElmer Spectrum Two spectrometer. Thermogravimetric analysis (TGA) was carried out on a Mettler-Toledo 2-HT instrument under nitrogen, with a heating rate of 10 °C min⁻¹ from room temperature to 1100 °C. X-ray photoelectron spectroscopy (XPS) measurements were performed on a PHI VersaProbe 4 system using Al Kα radiation. Elemental analysis was conducted with an Elementar UNICUBE analyzer. The C, H, and N contents were directly determined by elemental analysis. The O content was also directly measured using the same elemental analyzer, rather than being calculated by difference. For oxygen analysis, the sample was thermally decomposed at 1100 °C, and the generated oxygen-containing gaseous species, such as CO and CO₂, were analyzed to determine the oxygen content. Transmission electron microscopy (TEM) images were obtained using a Tecnai F20 G2 microscope operated at 120 kV.

**S2. Thermodynamic and mechanistic considerations of ammonia-based nitrogenation**

**S2.1. Thermodynamic estimation of representative nitrogenation pathways on graphene oxide**

To evaluate the thermodynamic feasibility of ammonia-based nitrogenation of graphene oxide (GO), we considered representative transformations associated with common oxygen-containing functionalities and calculated the standard enthalpy change (ΔH°), entropy change (ΔS°), and Gibbs free energy change (ΔG°) at 298 K. Reaction enthalpy and entropy changes were calculated from standard-state thermochemical data according to:

Δ_r_H° = $\sum_{products} \nu$ $H^{\circ}$_m_− $\sum_{reactants} \nu H^{\circ}$_m_

Δ_r_S° = $\sum_{products} \nu$ S°_m_− $\sum_{reactants} \nu S^{\circ}$_m_

Δ_r_G° = Δ_r_H° − T Δ_r_S° (T = 298 K)

where ν is the stoichiometric coefficient of each species.

For acid–base equilibrium:

ΔG° = − R T lnK

where R is the universal gas constant (8.314 J mol^-1^ K^-1^), T is the absolute temperature (K), and K is the equilibrium constant.

| **Table S1.** Model reactions and calculated thermodynamic parameters (298 K)*^a^* | | | | |
| --- | --- | --- | --- | --- |
|  | Model reaction | **ΔH°**  **(kJ mol⁻¹)** | **ΔS°**  **(J mol⁻¹ K⁻¹)** | ΔG°  (kJ mol⁻¹) |
| **R1** | C–OH + NH₃ → C–NH₂ + H₂O | −48.79 | −99.81 | −19.04 |
| **R2** | oxirane + NH₃ → 2-aminoethanol | −408.92 | −115.77 | −374.40 |
| **R3** | CH₃COOH + NH₃ → CH₃CONH₂ + H₂O | −71.39 | -165.82 | −21.94 |
| **R4** | CH₃COOH + NH₃ → CH₃COO⁻ + NH₄⁺ | - | - | −25.63  (K = 3.09×10⁴) |
| **R5** | CH₃COOH + NH₄⁺ + OH⁻ →CH₃COONH₄ + H₂O | - | - | −52.70  (K ≈ 1.74×10⁹) |

*^a^* Standard thermodynamic data were obtained from the National Institute of Standards and Technology (NIST) unless otherwise specified.

**S2.2. Kinetic considerations: why direct conversion of C–OH to C–NH_2_ is sluggish**

The model alcohol amination reaction (R1) can be thermodynamically favorable but is generally kinetically sluggish without catalysis or any prior activation of the alcohol. Direct substitution requires loss of OH⁻ (a poor leaving group) and cleavage of a strong C–O bond; also, with NH₃ solvated in water, kinetic barriers for amine formation increase because strong solvation stabilizes the hydroxyl group, increasing the activation energy for nucleophilic attack. Additionally, the ammonium ion lacks nucleophilicity, and water promotes competitive hydrolysis of intermediates, hindering the reaction. Consequently, appreciable conversion typically requires C–O activation (e.g., conversion of –OH to a better leaving group) or catalytic pathways that bypass direct substitution,^5^ such as dehydrogenation–imine formation–hydrogenation (“borrowing hydrogen”) sequences.^6^

Note for R5, which is thermodynamically equivalent to the net ionic neutralization CH₃COOH + OH⁻ → CH₃COO⁻ + H₂O, where NH₄⁺ is a spectator ion (present in both reactant and product as the ammonium counterion). The equilibrium constant was estimated from:

K = $\frac{Ka}{Kw}$ = 10^(pKw – pKa)^

using pK_a_(CH₃COOH) = 4.76 and pK_w_ = 14.00 at 298 K, giving K ≈ 1.74 × 10⁹ and ΔG° = −RT lnK ≈ −52.7 kJ mol⁻¹.

Overall, R1, R2, R4, and R5 show negative ΔG° values, indicating that these pathways are thermodynamically favorable under standard conditions and feasible under hydrothermal environments. Although the calculated ΔG° for amide formation (R3) is also negative, the large negative ΔS° and the dehydration requirement imply a higher kinetic barrier under typical hydrothermal conditions; thus, direct amidation is expected to be less competitive at the temperatures explored here, consistent with common textbook/literature descriptions that such condensations generally require higher temperatures (around 300°C) to proceed efficiently.^7, 8^

**S3. Supplementary Characterization**

**S3.1. XRD analysis**

XRD patterns (Figure 1f) reveal the structural evolution of the samples. GO exhibits a (001) diffraction peak at 2θ ≈ 9.8°, while rGO shows a broad (002) reflection near 26°, consistent with partial restacking after reduction. In contrast, both rCGO and NrCGO display no considerable layered diffraction features, confirming that the crumpled morphology effectively suppresses long-range stacking and preserves a non-layered structure after nitrogenation.^9^ This result is consistent with our previous report that rCGO possesses an anisotropic and non-layered framework.^2^ The absence of obvious diffraction changes after nitrogenation further indicates that the vapor-phase hydrothermal process preserves the crumpled framework without inducing structural collapse or graphitic restacking.

**S3.2. FTIR characterization**

FTIR spectra (Figure 1g) provided further insight into the evolution of surface chemistry during nitrogenation. The precursor CGO exhibited characteristic oxygen-containing functional groups, including a broad O–H stretching band around 3400 cm⁻¹, a carbonyl C=O stretching vibration at ~1720 cm⁻¹, C=C skeletal vibration near ~1620 cm⁻¹, C–OH vibration around ~1380 cm⁻¹, epoxy-related C–O vibration at ~1180 cm⁻¹, and tertiary alcohol C–OH stretching at ~1040 cm⁻¹, consistent with typical graphene oxide derivatives.^10-12^ For the blank control sample (rCGO), the O–H and C=O vibrations became substantially weakened after hydrothermal treatment, indicating partial removal of oxygen-containing functionalities. In contrast, NrCGO retained attenuated oxygen-related bands together with the appearance of a new absorption band at ~1420 cm⁻¹, attributable to C–N stretching vibrations.^13, 14^ The emergence of this feature provides spectroscopic evidence for nitrogen incorporation into the crumpled graphene framework.

**S3.3. Raman analysis**

Raman spectra (Figure 1h) of rCGO and NrCGO display the characteristic D (~1350 cm⁻¹) and G (~1580 cm⁻¹) bands associated with graphitic carbon materials.^9^ The I_D_/I_G_ ratio does not show significant variation after nitrogenation, suggesting that the hydrothermal nitrogenation process does not substantially alter the overall defect density or graphitic framework.^15^ These results further support that the crumpled graphene framework remains largely preserved during nitrogen incorporation.

**S3.4. TG analysis**

Thermogravimetric analysis (TGA, Figure 1i) was conducted to evaluate the thermal stability and decomposition behavior of rCGO and NrCGO. rCGO exhibits a typical decomposition profile for reduced crumpled graphene oxide.^16^ An initial weight loss of 3.41 wt% below 100 °C is attributed to desorption of physically adsorbed water.^17^ A subsequent weight decrease of 1.96 wt% between 100 and 200 °C corresponds to the decomposition of residual hydroxyl and epoxy functionalities.^18-20^ Owing to the partially reduced nature of rCGO, this low-temperature weight loss is considerably smaller than that commonly observed for graphene oxide.^2^

Between 200 and 750°C, rCGO undergoes a gradual weight loss of 14.24 wt%, mainly associated with the decomposition of residual carboxyl, carbonyl, and ether functionalities. A further pronounced decrease of 16.79 wt% at 750–900°C is attributed to the decomposition of thermally stable ether-related structures.^17, 21^ The remaining mass gradually approaches a stable plateau near 1100°C, reflecting the intrinsic thermal stability of the reduced graphene framework.

In contrast, NrCGO exhibits a more pronounced weight loss beginning at approximately 200°C. This additional decomposition is associated with thermally unstable nitrogen-containing functionalities together with residual oxygen-containing groups. Between 300 and 1000°C, NrCGO continues to exhibit greater weight loss than rCGO, consistent with the gradual decomposition of more thermally stable nitrogen configurations, including pyrrolic and pyridinic nitrogen species, along with residual oxygen functionalities. Above 1000°C, the slow and continuous weight loss is consistent with the decomposition of highly stable graphitic nitrogen species.^3, 22, 23^

Overall, the relatively limited weight loss below 300°C, compared with the substantial decomposition at higher temperatures, suggests that a significant fraction of the incorporated nitrogen exists in thermally stable doped configurations within the carbon framework rather than solely as weakly bound surface-grafted species.

**S3.5. BET/BJH analysis**

N₂ adsorption–desorption isotherms (Figure 1j) reveal that both rCGO and NrCGO exhibit macroporous-type adsorption behavior without obvious hysteresis, consistent with our previous observations for crumpled graphene oxide structures.^2^ Nitrogenation does not alter the overall pore type but leads to a decrease in BET surface area from 345.7 to 206.0 m² g⁻¹. This reduction is consistent with densification of the crumpled framework during nitrogenation, in agreement with the observed particle contraction and increased tap density. The BJH pore size distribution curves (Figure 1k) further support this interpretation. Both samples retain broad meso/macroporous distributions, while NrCGO exhibits a lower total pore volume (1.14 cm³ g⁻¹) than rCGO (1.40 cm³ g⁻¹), suggesting tighter packing within the crumpled structure after nitrogen incorporation. Overall, the BET and BJH analyses indicate that vapor-phase nitrogenation preserves the porous crumpled framework while inducing moderate structural densification.

**S3.6. XPS characterization and elemental distribution**

XPS spectra (Figure 2a,b) were collected to investigate the surface composition and nitrogen bonding configurations of rCGO and NrCGO. The survey spectra reveal that rCGO mainly contains C 1s (≈ 284.8 eV) and O 1s (≈ 532.2 eV) signals, whereas an additional N 1s peak appears at ≈ 400 eV after nitrogenation, confirming successful nitrogen incorporation into the crumpled graphene framework. Quantitative analysis shows that the N/C atomic ratio of NrCGO reaches approximately 0.24, significantly higher than values commonly reported for nitrogen-doped graphene synthesized under comparable hydrothermal or solvothermal conditions.

High-resolution N 1s deconvolution (Figure 2b) resolves four major components centered at 398, 400, 401, and 403 eV, corresponding to pyridinic N, pyrrolic N, ammonium N, and oxidized N species, respectively.^24-26^ It should be noted that pyrrolic N and amine-like nitrogen species appear in very similar N 1s binding-energy regions; therefore, the component at 400 eV may also include contributions from amine-like N species. In addition, although graphitic-N is commonly observed near 401 eV in highly thermally treated nitrogen-doped carbons, the formation of true graphitic-N through direct substitution into intact graphitic domains is unlikely under the relatively mild hydrothermal treatment conditions used in this work. Therefore, the 401 eV component is more cautiously assigned to ammonium-like/protonated N species rather than graphitic-N.^27^ Overall, the N 1s results suggest that nitrogen incorporation mainly occurs at defect-associated, edge-like, or oxygen-functional-group-associated sites rather than through predominant graphitic substitution.^3^

The HAADF-STEM image (Figure 2c), together with the corresponding EDS elemental maps (Figure 2d-f), further reveals homogeneous spatial distributions of C, O, and N throughout the crumpled framework, indicating uniform nitrogen incorporation within the nitrogenated crumpled graphene structure.

**Figure S1.** High-resolution C 1s XPS spectra and peak deconvolution of (a) 3×GO, (b) 1×GO, and (c) commercial GO. The fitted components are assigned to C–C(sp²), C–C(sp³), C–OH, C–O–C, C=O, and O–C=O species. The relative contribution of the C–O–C component increases with increasing oxidation degree, supporting the enhanced abundance of epoxide-related functionalities in highly oxidized GO.

**Figure S2.** Comparative IR spectra of NrCGO and thermally reduced NrCGO samples treated at 300, 600, and 900 °C.

**Figure S3.** The LSV curves of rCGO and NrCGO-600°C in O_2_-saturated 0.1 M KOH.

| **Table S2.** Elemental compositions of NrCGO synthesized under different hydrothermal reaction conditions, determined by elemental analysis. | N  (wt%) | 12.9 ± 0.7 | 13.7 ± 2.8 | 13.2 ± 1.0 | 11.8 ± 1.8 |  |
| --- | --- | --- | --- | --- | --- | --- |
|  | O  (wt%) | 6.5 ± 0.2 | 6.3 ± 3.0 | 5.3 ± 1.3 | 5.4 ± 2.3 |  |
|  | C  (wt%) | 72.9 ± 0.8 | 72.0 ± 0.3 | 72.7 ± 1.2 | 74.1 ± 0.9 |  |
|  | NH₃ _(aq)_ volume  (mL) | 2.5 | 5 | 10 | 20 |  |
|  |  |  |  |  |  |  |
|  | N  (wt%) | 10.9 ± 1.5 | 13.2 ± 1.0 | 13.2 ± 2.3 | 13.4 ± 0.4 |  |
|  | O  (wt%) | 5.0 ± 2.8 | 5.3 ± 1.3 | 10.9 ± 0.1 | 10.0 ± 1.6 |  |
|  | C  (wt%) | 74.3 ± 3.5 | 72.7 ± 1.2 | 75.3 ± 2.6 | 75.2 ± 2.7 |  |
|  | Reaction time (h) | 6 | 12 | 18 | 24 |  |
|  |  |  |  |  |  |  |
|  | N  (wt%) | 11.1 ± 0.3 | 12.0 ± 1.0 | 13.2 ± 3.2 | 13.2 ± 0.5 | 12.3 ± 0.9 |
|  | O  (wt%) | 6.4 ± 0.5 | 8.6 ± 0.4 | 5.3 ± 1.3 | 5.8 ± 1.5 | 3.3 ± 1.6 |
|  | C  (wt%) | 73.2 ± 0.6 | 72.5 ± 4.0 | 72.7 ± 2.3 | 75.0 ± 0.6 | 67.6 ± 2.8 |
|  | Reaction  temp. (˚C) | 140 | 160 | 180 | 190 | 200 |

| **Table S3.** Elemental compositions of NrCGO synthesized using graphene oxide precursors with different oxidation degrees and structures, determined by elemental analysis. | | | | | | | | |
| --- | --- | --- | --- | --- | --- | --- | --- | --- |
| Oxidation degree of GO (O/C) | C (wt%) | O (wt%) | N (wt%) |  | GO structure | C (wt%) | O (wt%) | N (wt%) |
| 0.41 | 72.7 ± 1.2 | 5.3 ± 1.3 | 13.2 ± 1.0 |  | GO | 76.4 ± 2.0 | 6.2 ± 1.9 | 13.7 ± 0.4 |
| 0.49 | 72.1 ± 0.2 | 6.6 ± 0.1 | 12.6 ± 0.2 |  | CGO | 72.7 ± 1.2 | 5.3 ± 1.3 | 13.2 ± 1.0 |
| 0.55 | 59.5 ± 3.9 | 10.6 ± 2.7 | 16.5 ± 1.1 |  |  |  |  |  |

| **Table S4.** Elemental compositions of NrCGO synthesized using different nitrogen sources and after annealing at different temperatures, determined by elemental analysis. | | | | | | | | |
| --- | --- | --- | --- | --- | --- | --- | --- | --- |
| N-source | C (wt%) | O (wt%) | N (wt%) |  | Annealing temp. (˚C) | C (wt%) | O (wt%) | N (wt%) |
| NH_3_ | 59.5±3.9 | 10.6$\pm$2.7 | 16.5$\pm$1.1 |  | 300 | 62.1 | 18.6 | 14.4 |
| CH_3_NH_2_ | 62.0±0.9 | 6.1±0.5 | 9.99$\pm$0.8 |  | 600 | 71.6 | 14.9 | 9.02 |
| EDA | 60.9±1.3 | 6.1±1.5 | 14.1±0.3 |  | 900 | 80.7 | 11.1 | 6.30 |

| **Table S5.** Abundance percentages of different functional groups on the surfaces of commercial GO, 1$\times$GO, and 3$\times$GO based on high-resolution C 1s XPS peak deconvolution. | | | | | | |
| --- | --- | --- | --- | --- | --- | --- |
| GO material | O=C-O | C=O | C-O-C | C-OH | C-C (sp^3^) | C-C (sp^2^) |
| Commercial GO | 3.6 | 10.7 | 28.9 | 12.1 | 17.3 | 27.4 |
| 1$\times$GO | 4.1 | 13.3 | 30.8 | 12.3 | 16.2 | 23.3 |
| 3$\times$GO | 4.4 | 8.9 | 38.9 | 9.0 | 14.2 | 24.6 |

| **Table S6.** Comparison of Nitrogen Incorporation Levels in Reported Nitrogenated Graphene-Based Materials | | | | |
| --- | --- | --- | --- | --- |
| Graphene precursor | N-source | Method | N (at%) | Ref. |
| GO | Ammonia | Hydrothermal | 17.3 | This work |
| GO | Ammonia | Hydrothermal | 3.2 | [28] |
| GO | Ammonia + N_2_H_4_ | Hydrothermal | 4.1 | [29] |
| GO | Ammonia | Hydrothermal | 6.0 | [30] |
| GO | Urea | Hydrothermal | 6.6 | [31] |
| GO | Ammonia | Hydrothermal | 9.6 | [32] |
| GO | Urotropin | Hydrothermal | 5.8 | [33] |
| GO | NH_3_·H_2_O | Hydrothermal | 6.7 | [34] |
| GO | Ammonia | NH_3_ plasma | 7.3 | [35] |
| Acetonitrile | Acetonitrile | CVD | 8.3 | [36] |
| Acetylene | N_2_ | plasma-assisted CVD | 3.5 | [37] |
| GO | Ammonium thiocyanate | Thermal annealing | 11.9 | [38] |

| **Table S7.** Quantification of nitrogen configurations by TPD–MS and comparison among reported nitrogen-doped graphene/carbon materials | | | | |
| --- | --- | --- | --- | --- |
| Sample | Nitrogen configuration | | | Ref. |
|  | Pyridinic N% | Pyrrolic N% | Graphitic N% |  |
| **NrCGO-600°C** | **57%** | **32%** | **10%** | **This work** |
| Nitrogen-Doped Holey Carbon@Graphene | ~50% | ~25% | ~25% | [39] |
| NCG-1000 | 22.5% | 15.1% | 62.4% | [40] |
| PANi/RG-O | 48.6% | 44.7% | 6.7% | [41] |
| GN3 | 44% | 49% | 7% | [42] |
| UR-NG | 4.5% | 92.5% | 2.8% | [43] |

**References:**

(1) Chen, C.-H.; Hu, S.; Shih, J.-F.; Yang, C.-Y.; Luo, Y.-W.; Jhang, R.-H.; Chiang, C.-M.; Hung, Y., Jr. Effective Synthesis of Highly Oxidized Graphene Oxide That Enables Wafer-scale Nanopatterning: Preformed Acidic Oxidizing Medium Approach. *Sci. Rep.* **2017**, *7*, 3908.

(2) Liu, Y.-P.; Tang, W.-R.; Liu, Y.-T.; Devi, Y.; Chiou, K.; Chen, C.-H. Cryogenic synthesis of crumpled graphene Spheres: Advancing scalability, stability and preserved chemical versatility. *Carbon* **2025**, *242*, 120435.

(3) Yoshii, T.; Nishikawa, G.; Prasad, V. K.; Shimizu, S.; Kawaguchi, R.; Tang, R.; Chida, K.; Sato, N.; Sakamoto, R.; Takatani, K.; et al. Quantitative and qualitative analysis of nitrogen species in carbon at the ppm level. *Chem* **2024**, *10*, 2450-2463.

(4) Chastain, J.; King Jr, R. C. Handbook of X-ray photoelectron spectroscopy. *Perkin-Elmer Corporation* **1992**, *40*, 25.

(5) Yang, Q.; Wang, Q.; Yu, Z. Substitution of alcohols by N-nucleophiles via transition metal-catalyzed dehydrogenation. *Chem. Soc. Rev.* **2015**, *44*, 2305-2329.

(6) Corma, A.; Navas, J.; Sabater, M. J. Advances in One-Pot Synthesis through Borrowing Hydrogen Catalysis. *Chem. Rev.* **2018**, *118*, 1410-1459.

(7) Lundberg, H.; Tinnis, F.; Selander, N.; Adolfsson, H. Catalytic amide formation from non-activated carboxylic acids and amines. *Chem. Soc. Rev.* **2014**, *43*, 2714-2742.

(8) Montalbetti, C. A. G. N.; Falque, V. Amide bond formation and peptide coupling. *Tetrahedron* **2005**, *61*, 10827-10852.

(9) Yeh, C.-N.; Huang, H.; Lim, A. T. O.; Jhang, R.-H.; Chen, C.-H.; Huang, J. Binder-free graphene oxide doughs. *Nat. Commun.* **2019**, *10*, 422.

(10) Brusko, V.; Khannanov, A.; Rakhmatullin, A.; Dimiev, A. M. Unraveling the infrared spectrum of graphene oxide. *Carbon* **2024**, *229*, 119507.

(11) Rabchinskii, M. K.; Ryzhkov, S. A.; Besedina, N. A.; Brzhezinskaya, M.; Malkov, M. N.; Stolyarova, D. Y.; Arutyunyan, A. F.; Struchkov, N. S.; Saveliev, S. D.; Diankin, I. D.; et al. Guiding graphene derivatization for covalent immobilization of aptamers. *Carbon* **2022**, *196*, 264-279.

(12) Shnitov, V. V.; Rabchinskii, M. K.; Brzhezinskaya, M.; Stolyarova, D. Y.; Pavlov, S. V.; Baidakova, M. V.; Shvidchenko, A. V.; Kislenko, V. A.; Kislenko, S. A.; Brunkov, P. N. Valence Band Structure Engineering in Graphene Derivatives. *Small* **2021**, *17*, 2104316.

(13) Kao, W.-Y.; Chen, W.-Q.; Chiu, Y.-H.; Ho, Y.-H.; Chen, C.-H. General Solvent-dependent Strategy toward Enhanced Oxygen Reduction Reaction in Graphene/Metal Oxide Nanohybrids: Effects of Nitrogen-containing Solvent. *Sci. Rep.* **2016**, *6*, 37174.

(14) Bosch-Navarro, C.; Coronado, E.; Martí-Gastaldo, C. Controllable coverage of chemically modified graphene sheets with gold nanoparticles by thermal treatment of graphite oxide with N,N-dimethylformamide. *Carbon* **2013**, *54*, 201-207.

(15) Brzhezinskaya, M.; Kapitanova, O. O.; Kononenko, O. V.; Koveshnikov, S.; Korepanov, V.; Roshchupkin, D. Large-scalable graphene oxide films with resistive switching for non-volatile memory applications. *J. Alloys Compd.* **2020**, *849*, 156699.

(16) Liu, W.; Speranza, G. Tuning the Oxygen Content of Reduced Graphene Oxide and Effects on Its Properties. *ACS Omega* **2021**, *6*, 6195-6205.

(17) Eigler, S.; Dotzer, C.; Hirsch, A.; Enzelberger, M.; Müller, P. Formation and Decomposition of CO2 Intercalated Graphene Oxide. *Chem. Mater.* **2012**, *24*, 1276-1282.

(18) Acik, M.; Lee, G.; Mattevi, C.; Chhowalla, M.; Cho, K.; Chabal, Y. J. Unusual infrared-absorption mechanism in thermally reduced graphene oxide. *Nat. Mater.* **2010**, *9*, 840-845.

(19) Acik, M.; Lee, G.; Mattevi, C.; Pirkle, A.; Wallace, R. M.; Chhowalla, M.; Cho, K.; Chabal, Y. The Role of Oxygen during Thermal Reduction of Graphene Oxide Studied by Infrared Absorption Spectroscopy. *J. Phys. Chem. C* **2011**, *115*, 19761-19781.

(20) Klemeyer, L.; Park, H.; Huang, J. Geometry-Dependent Thermal Reduction of Graphene Oxide Solid. *ACS Mater. Lett.* **2021**, *3*, 511-515.

(21) Tang, H.; Ehlert, G. J.; Lin, Y.; Sodano, H. A. Highly Efficient Synthesis of Graphene Nanocomposites. *Nano Lett.* **2012**, *12*, 84-90.

(22) Das, N. K.; Santra, S.; Naik, P. K.; Vasa, M. S.; Raj, R.; Bose, S.; Banerjee, T. Evaluation of Thermophysical Properties and Thermal Performance of Amine-Functionalized Graphene Oxide/Deep Eutectic Solvent Nanofluids as Heat-Transfer Media for Desalination Systems. *ACS Sustainable Chem. Eng.* **2023**, *11*, 5376-5389.

(23) Gangopadhyay, B.; Mallik, A.; Ali, M. S.; Halder, A.; Layek, R.; Karmakar, D.; Karmakar, S.; Dutta, K.; Das, T. D.; Panda, S.; et al. Thermal Effects on Electrochemical Performance of Copper Oxide Nanoparticles Decorated Amine-Functionalized Graphene Oxide for Ultrahigh Energy Density Supercapacitor with Real-Life Application. *J. Phys. Chem. C* **2023**, *127*, 21940-21953.

(24) Guo, D.; Shibuya, R.; Akiba, C.; Saji, S.; Kondo, T.; Nakamura, J. Active sites of nitrogen-doped carbon materials for oxygen reduction reaction clarified using model catalysts. *Science* **2016**, *351*, 361-365.

(25) Zorn, G.; Liu, L.-H.; Árnadóttir, L.; Wang, H.; Gamble, L. J.; Castner, D. G.; Yan, M. X-ray Photoelectron Spectroscopy Investigation of the Nitrogen Species in Photoactive Perfluorophenylazide-Modified Surfaces. *J. Phys. Chem. C* **2014**, *118*, 376-383.

(26) Pels, J. R.; Kapteijn, F.; Moulijn, J. A.; Zhu, Q.; Thomas, K. M. Evolution of nitrogen functionalities in carbonaceous materials during pyrolysis. *Carbon* **1995**, *33*, 1641-1653.

(27) Ferré-Pujol, P.; Obata, S.; Raya, J.; Bianco, A.; Katayama, H.; Kato, T.; Nishina, Y. Reversible chemical modifications of graphene oxide for enhanced viral capture and release in water. *Carbon* **2025**, *234*, 120015.

(28) Kumar, N. A.; Nolan, H.; McEvoy, N.; Rezvani, E.; Doyle, R. L.; Lyons, M. E. G.; Duesberg, G. S. Plasma-assisted simultaneous reduction and nitrogen doping of graphene oxide nanosheets. *J. Mater. Chem. A* **2013**, *1*, 4431-4435.

(29) Long, D.; Li, W.; Ling, L.; Miyawaki, J.; Mochida, I.; Yoon, S.-H. Preparation of Nitrogen-Doped Graphene Sheets by a Combined Chemical and Hydrothermal Reduction of Graphene Oxide. *Langmuir* **2010**, *26*, 16096-16102.

(30) Li, J.; Li, X.; Zhao, P.; Lei, D. Y.; Li, W.; Bai, J.; Ren, Z.; Xu, X. Searching for magnetism in pyrrolic N-doped graphene synthesized via hydrothermal reaction. *Carbon* **2015**, *84*, 460-468.

(31) Guo, H.-L.; Su, P.; Kang, X.; Ning, S.-K. Synthesis and characterization of nitrogen-doped graphene hydrogels by hydrothermal route with urea as reducing-doping agents. *J. Mater. Chem. A* **2013**, *1*, 2248-2255.

(32) Chen, P.; Yang, J.-J.; Li, S.-S.; Wang, Z.; Xiao, T.-Y.; Qian, Y.-H.; Yu, S.-H. Hydrothermal synthesis of macroscopic nitrogen-doped graphene hydrogels for ultrafast supercapacitor. *Nano Energy* **2013**, *2*, 249-256.

(33) Zou, Y.; Kinloch, I. A.; Dryfe, R. A. W. Nitrogen-doped and crumpled graphene sheets with improved supercapacitance. *J. Mater. Chem. A* **2014**, *2*, 19495-19499.

(34) Ma, Z.; Huang, X.; Dou, S.; Wu, J.; Wang, S. One-Pot Synthesis of Fe2O3 Nanoparticles on Nitrogen-Doped Graphene as Advanced Supercapacitor Electrode Materials. *J. Phys. Chem. C* **2014**, *118*, 17231-17239.

(35) Hassan, F. M.; Chabot, V.; Li, J.; Kim, B. K.; Ricardez-Sandoval, L.; Yu, A. Pyrrolic-structure enriched nitrogen doped graphene for highly efficient next generation supercapacitors. *J. Mater. Chem. A* **2013**, *1*, 2904-2912.

(36) Tang, R.; Aziz, A.; Yu, W.; Pan, Z.-Z.; Nishikawa, G.; Yoshii, T.; Nomura, K.; Taylor, E. E.; Stadie, N. P.; Inoue, K.; et al. Prominent Structural Dependence of Quantum Capacitance Unraveled by Nitrogen-Doped Graphene Mesosponge. *Small* **2024**, *20*, 2308066.

(37) Zhai, Z.; Shen, H.; Chen, J.; Li, X.; Jiang, Y. Direct growth of nitrogen-doped graphene films on glass by plasma-assisted hot filament CVD for enhanced electricity generation. *J. Mater. Chem. A* **2019**, *7*, 12038-12049.

(38) Du, D.; Li, P.; Ouyang, J. Nitrogen-Doped Reduced Graphene Oxide Prepared by Simultaneous Thermal Reduction and Nitrogen Doping of Graphene Oxide in Air and Its Application as an Electrocatalyst. *ACS Appl. Mater. Interfaces* **2015**, *7*, 26952-26958.

(39) Sun, J.; Lowe, S. E.; Zhang, L.; Wang, Y.; Pang, K.; Wang, Y.; Zhong, Y.; Liu, P.; Zhao, K.; Tang, Z.; et al. Ultrathin Nitrogen-Doped Holey Carbon@Graphene Bifunctional Electrocatalyst for Oxygen Reduction and Evolution Reactions in Alkaline and Acidic Media. *Angew. Chem. Int. Ed.* **2018**, *57*, 16511-16515.

(40) Wang, M.; Wang, J.; Hou, Y.; Shi, D.; Wexler, D.; Poynton, S. D.; Slade, R. C. T.; Zhang, W.; Liu, H.; Chen, J. N-Doped Crumpled Graphene Derived from Vapor Phase Deposition of PPy on Graphene Aerogel as an Efficient Oxygen Reduction Reaction Electrocatalyst. *ACS Appl. Mater. Interfaces* **2015**, *7*, 7066-7072.

(41) Lai, L.; Potts, J. R.; Zhan, D.; Wang, L.; Poh, C. K.; Tang, C.; Gong, H.; Shen, Z.; Lin, J.; Ruoff, R. S. Exploration of the active center structure of nitrogen-doped graphene-based catalysts for oxygen reduction reaction. *Energy Environ. Sci.* **2012**, *5*, 7936-7942.

(42) Šedajová, V.; Bakandritsos, A.; Błoński, P.; Medveď, M.; Langer, R.; Zaoralová, D.; Ugolotti, J.; Dzíbelová, J.; Jakubec, P.; Kupka, V.; et al. Nitrogen doped graphene with diamond-like bonds achieves unprecedented energy density at high power in a symmetric sustainable supercapacitor. *Energy Environ. Sci.* **2022**, *15*, 740-748.

(43) Sun, H.; Wang, Q.; Wu, T.; Miao, Y.; Fang, Y. Plasma-assisted synthesis of pyrrolic-nitrogen doped reduced graphene oxide to enhance supercapacitor performance. *Appl. Surf. Sci.* **2020**, *527*, 146574.
